# Internet-Based Cognitive Behavioral Therapy to Reduce Suicidal Ideation: A Systematic Review and Meta-analysis

**DOI:** 10.1001/jamanetworkopen.2020.3933

**Published:** 2020-04-28

**Authors:** Rebekka Büscher, Michelle Torok, Yannik Terhorst, Lasse Sander

**Affiliations:** 1 Department of Rehabilitation Psychology and Psychotherapy, University of Freiburg, Freiburg, Germany; 2 Black Dog Institute, University of New South Wales, Sydney, Australia; 3 Department of Research Methods, Institute of Psychology and Education, University of Ulm, Ulm, Germany; 4 Department of Clinical Psychology and Psychotherapy, Institute of Psychology and Education, University of Ulm, Ulm, Germany

## Abstract

**Importance:**

Suicidal ideation is a widespread phenomenon. However, many individuals at risk for suicide do not seek treatment, which might be addressed by providing low-threshold, internet-based self-help interventions.

**Objective:**

To investigate whether internet-based self-help interventions directly targeting suicidal ideation or behavior are associated with reductions in suicidal ideation.

**Data Sources:**

A systematic search of PsycINFO, MEDLINE, Cochrane Central Register of Controlled Trials (CENTRAL), and the Centre for Research Excellence of Suicide Prevention (CRESP) databases for trials from inception to April 6, 2019, was performed, supplemented by reference searches. Search strings consisted of various search terms related to the concepts of internet, suicide, and randomized clinical trials.

**Study Selection:**

Two independent researchers reviewed titles, abstracts, and full texts. Randomized clinical trials evaluating the effectiveness of internet-based self-help interventions to reduce suicidal ideation were included. Interventions were eligible if they were based on psychotherapeutic elements. Trials had to report a quantitative measure of a suicide-specific outcome. Mobile-based and gatekeeper interventions were excluded; no further restrictions were placed on participant characteristics or date of publication.

**Data Extraction and Synthesis:**

This study followed the Preferred Reporting Items for Systematic Reviews and Meta-analyses (PRISMA) reporting guidelines. Risk of bias was evaluated using the Cochrane Risk of Bias Tool. Standardized mean differences were calculated using a random-effects model.

**Main Outcomes and Measures:**

Suicidal ideation was the a priori primary outcome.

**Results:**

Six unique eligible trials (1567 unique participants; 1046 [66.8%] female; pooled mean [SD] age, 36.2 [12.5] years) were included in the systematic review and meta-analysis. All identified interventions were internet-based cognitive behavioral therapy (iCBT). Participants assigned to the iCBT condition experienced a significantly reduced suicidal ideation compared with controls following intervention in all 6 trials (standardized mean difference, −0.29; 95% CI, −0.40 to −0.19; *P* < .001). Heterogeneity was low (*I*^2^ = 0%). The effect appeared to be maintained at follow-up in 4 trials (standardized mean difference, −0.18; 95% CI, −0.34 to −0.02; *P* = .03; *I*^2^ = 36%). Studies did not report sufficient data on completed suicides and suicide attempts to assess potential associations.

**Conclusions and Relevance:**

These results show that iCBT interventions are associated with significant reductions in suicidal ideation compared with control conditions. Considering their high scalability, iCBT interventions have the potential to reduce suicide mortality. Future research should assess the effect of these digital health interventions on suicidal behavior and identify moderators and mediators to advance understanding of the mechanisms of effectiveness of these interventions.

## Key Points

**Question:**

Is internet-based cognitive behavioral therapy directly targeting suicidal ideation or behaviors associated with reduced suicidal ideation?

**Findings:**

In this meta-analysis including 6 unique randomized clinical trials and 1567 unique participants, internet-based cognitive behavioral therapy interventions for suicide prevention were associated with significantly reduced suicidal ideation after intervention compared with controls. First indications suggest that the treatment effect might be maintained at follow-up.

**Meaning:**

Internet-based self-help interventions for suicide prevention based on cognitive behavioral therapy may be effective in reducing suicidal ideation and may be considered as a low-threshold treatment option, complementing current services.

## Teaser

This systematic review and meta-analysis investigates whether internet-based self-help interventions directly targeting suicidal ideation or behavior are associated with reductions in suicidal ideation among patients in randomized clinical trials.

## Introduction

Suicidal ideation is a common phenomenon^1,2,3,4,5^ that often precedes suicide attempts and suicide deaths.^1^ Accordingly, suicidal ideation may be an important target for suicide prevention efforts, particularly as an indicator for early detection and intervention to avoid or reduce nonfatal and fatal outcomes.^6,7^ Cognitive behavioral therapy (CBT), including dialectical behavioral therapy, has been shown to be effective in the reduction of suicidal ideation and behaviors.^8^ Cognitive behavioral therapy is based on the assumption that cognitions play a central role in the development and maintenance of mental health issues and that emotional and behavioral problems can be resolved by modifying dysfunctional thoughts.^9^ Although effective treatments are available,^8,10,11^ many individuals at risk of suicide do not receive professional help.^12^ Barriers to treatment seeking in suicidal individuals include the wish to solve the problem by oneself, the belief that the problem is not that severe, stigma, limited access to treatment, and financial issues.^12^

There are high levels of technology use among suicidal persons.^13^ Moreover, suicidal persons are more likely to seek help online than in face-to-face settings,^14^ which might be due to the anonymity provided by digital resources.^15^ Thus, internet-based interventions might be an appropriate and low-threshold approach to address the barriers to treatment seeking.^15,16^

Growing interest in the applicability of digital technologies as scalable health solutions has seen stand-alone, internet-based self-help interventions (ISIs) become an emerging focus of health research in recent years.^15^ To date, most ISIs are based on CBT (internet-based CBT [iCBT]) because the highly structured and often manualized therapy elements can be transferred well into a digital format.^15^ The effectiveness of ISIs is well established for various mental disorders,^17,18,19,20^ and they have been integrated in clinical practice in several countries.^15,21^ However, ISIs for suicide prevention have not received the same attention. New studies using robust randomized trial designs have been published in recent years, warranting an update of the evidence.

Previous reviews in the field of digital interventions for suicide prevention have several limitations.^22,23,24,25,26^ First, most reviews have included a wide range of heterogeneous digital approaches for suicide prevention, such as websites, email support, online message boards, online support groups, and mobile interventions.^23,25,26^ The resulting statistical and clinical heterogeneity makes it difficult to draw valid conclusions on their effectiveness.^27,28^ Second, reviews often did not differentiate interventions directly addressing suicidality from those that focused on other conditions, such as depression.^23,24,26^ Third, study inclusion was often not restricted to randomized clinical trials (RCTs),^22,23,25,26^ which is methodologically inadequate because suicidal ideation seems to decrease even in control conditions.^22^ Fourth, risk of bias was not assessed in some cases.^22,25^ Fifth, previous reviews have typically not evaluated publication bias.^22,23,24,25^

The aim of this systematic review and meta-analysis is to examine whether ISIs that have been specifically designed to target suicidal ideation or behavior are associated with reductions in these outcomes. By addressing major methodological limitations of prior reviews, accurate evidence of the effects of ISIs is established in this review.

## Methods

### Search Strategy and Selection Criteria

This systematic review and meta-analysis was reported according to the Preferred Reporting Items for Systematic Reviews and Meta-analyses (PRISMA) reporting guideline.^29^ We searched the following databases from inception to April 6, 2019: PsycINFO, MEDLINE, Cochrane Central Register of Controlled Trials (CENTRAL), and the Centre for Research Excellence of Suicide Prevention (CRESP). Search strings combined a variety of search terms related to the concepts of internet, suicide, and RCT and can be viewed in the study protocol.^30^ We conducted a pilot testing of the search strategy using a validation set of 5 eligible RCTs. All of these trials were identified using the search strategy. In addition, we screened the reference lists of all included studies and relevant review articles for additional studies (backward search), and we screened studies that cited the included studies and relevant reviews (forward search).^28^ Following this, we conducted a search on ClinicalTrials.gov on June 19, 2019, to examine publication bias and identify ongoing trials that are presented to give an overview of current developments in the field, but these results were not included in the meta-analysis. We did not search for gray literature.

Studies were eligible, first, if they used ISIs that directly targeted suicidal ideation or behaviors, were delivered predominantly in an online setting, and were based on psychological elements. Second, control groups could receive treatment as usual, another active or passive treatment, placebo, or no intervention or consist of a wait-list group. Third, studies had to report a quantitative measure of a suicide-specific outcome (ie, suicidal ideation, completed suicide, or suicide attempt). Fourth, eligible RCTs were provided in English or German and available in full text. Studies were excluded if they used mobile-based interventions or interventions that targeted gatekeepers (eg, teachers). We did not place any restrictions on sociodemographic characteristics of participants or date of publication.

**Figure 1.**
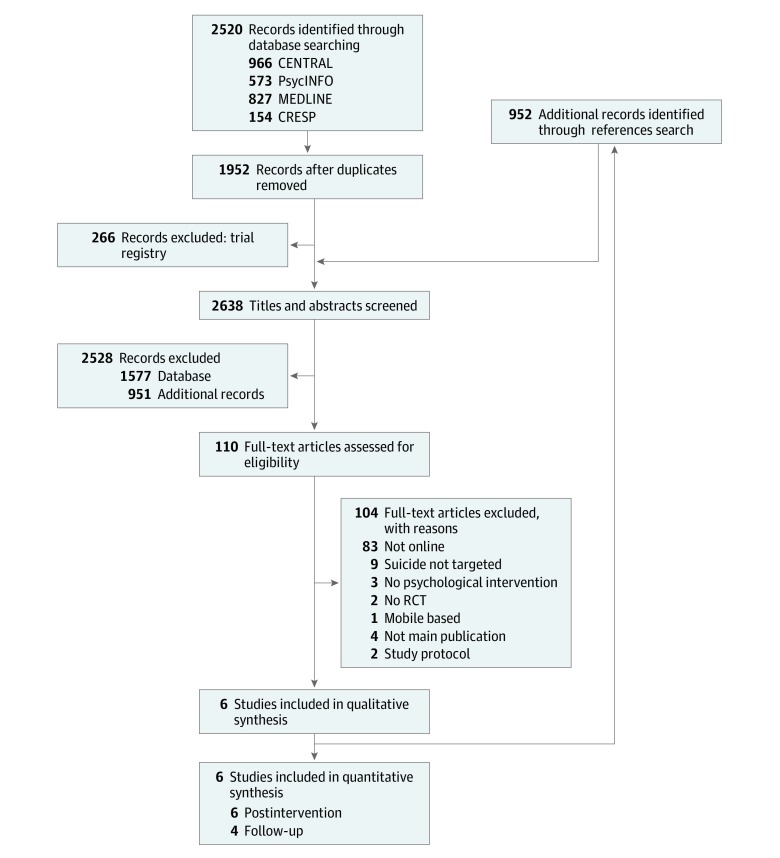
PRISMA Flowchart of Included Studies CENTRAL indicates Cochrane Central Register of Controlled Trials; CRESP, Centre for Research Excellence of Suicide Prevention; and RCTs, randomized clinical trials.

We analyzed summary data that were reported in the study or provided by authors. Two reviewers (R.B. and M.T.) independently screened the studies for eligibility in a hierarchical approach: in a first step, they screened titles and abstracts identified in the databases. In a second step, they screened full-text articles. Discrepancies were resolved in discussion with a third researcher (L.S.). The selection process is displayed in the PRISMA flowchart (Figure 1).

The procedure was predefined and described in detail in a study protocol.^30^ We registered the study with the International Prospective Register of Systematic Reviews (CRD42019130253). Amendments to the protocol are displayed in the eMethods in the Supplement.

### Data Extraction

We used an investigator-developed data extraction form. Data were extracted by 1 researcher (R.B.) and double-checked by a second researcher (M.T. or Y.T.). The following data were extracted from the included studies: study identification items, study design, description of the intervention and control conditions, technical characteristics, population, setting, treatment adherence, study dropout, outcome variables, and results. If relevant data were not reported, we contacted corresponding authors via email to provide them. Authors of 2 eligible studies were asked to provide data that we received for 1 study. Where available, we used intent-to-treat data, including data from all randomized participants. To avoid bias through missing data, we extracted data from imputation models or estimated in robust multilevel analyses. The effect measures included in the meta-analysis were unadjusted. Suicidal ideation was the primary outcome. The following variables were included as secondary outcomes: suicide and suicide attempt, depressiveness, anxiety, and hopelessness. If multiple measures were used, we prioritized data extraction as follows: (1) validated questionnaires, (2) clinician ratings, and (3) single-item analysis of other rating scales.

### Risk of Bias and Quality of Evidence

The risk of bias was evaluated with the Cochrane Risk of Bias tool that assesses risk of selection bias, performance bias, detection bias, attrition bias, reporting bias, and other bias.^31^ The quality of evidence for the primary outcome was evaluated according to the Grading of Recommendations Assessment, Development, and Evaluation (GRADE).^32^ The GRADE assessments include the domains risk of bias, inconsistency, indirectness of evidence, imprecision of the estimate, and publication bias on outcome level. Both risk of bias and the quality of evidence were assessed by 2 independent researchers (R.B. and M.T.). Potential discrepancies were resolved in discussion with a third researcher (L.S.).

To determine whether publication bias was likely, we inspected an international trial registry (ClinicalTrials.gov) and used the systematic database searches to identify relevant trial protocols. Afterwards, we checked for subsequent publications.

### Statistical Analysis

We conducted a random-effects meta-analysis for the primary outcome post intervention and at medium-term follow-up. We estimated standardized mean differences (SMDs) using Hedges *g*^28^ and respective 95% CIs; it was a between-group effect of mean changes from baseline, standardized by the pooled SD at baseline. Statistical heterogeneity was analyzed using *I*^2^ statistics. According to the GRADE handbook, an *I*^2^ statistic of less than 40% indicates low heterogeneity; 30% to 60%, moderate; 50% to 90%, substantial; and 75% to 100%, considerable.^32^ We conducted a preplanned subgroup analysis for different types of control groups (active vs wait-list controls). We conducted predefined sensitivity analyses by only including interventions for adults and by only including unguided interventions. Unguided ISIs were defined as interventions that do not involve any human support, whereas in guided ISI, a therapist provides feedback and guidance in addition to the self-help material that can be used independently.^15^ Another predefined sensitivity analysis was conducted by excluding trials at high risk of bias (ie, >2 items of the Cochrane Risk of Bias Tool^31^ rated at high risk). We performed a post hoc sensitivity analysis by excluding a trial that had a high weight (43.3%) on the pooled effect size. All subgroup and sensitivity analyses were only performed for the effects post intervention.

Effects on secondary outcomes were reported narratively. The Cochrane Collaboration’s Review Manager (RevMan, version 5.3) was used for calculation. We used R, version 3.6.1 (R Project for Statistical Computing), for preparatory analyses. Two-sided *P* < .05 indicated statistical significance.

## Results

### Selection, Inclusion, and Study Characteristics

We identified 2638 records for screening. A total of 6 unique studies^33,34,35,36,37,38^ (1567 unique baseline participants; 1046 [66.8%] female; pooled mean [SD] age, 36.2 [12.5] years) were included in the systematic review and meta-analysis. The study selection process is displayed along with reasons for exclusion in Figure 1.

A summary of included studies is provided in the Table. All studies were published from 2014 to 2019. Two interventions^34,35^ were designed for youth (mean [SD] age, 16.9 [1.7] and 14.7 [1.4] years), whereas 4 interventions^33,36,37,38^ were designed for adults (mean [SD] age range, 14.7 [1.4] to 40.9 [13.7] years). The proportion of female participants ranged from 59.4% to 82.0%. All interventions were developed for the reduction of preexisting suicidal ideation or behaviors and were based on iCBT, including third-wave therapies such as dialectical behavioral therapy. Five studies^33,34,36,37,38^ reported using homework or exercises in their programs; only 2 interventions were guided.^35,37^ Exposure time ranged from 2 weeks^34^ to 10 weeks.^35^ Most of the trials^33,35,36,37,38^ reported safety procedures for enrolled participants. This involved contacting participants who reported suicidal ideation above a predefined threshold, and in 2 trials,^33,38^ the participant’s psychiatrist or general clinician was called when the risk threshold defined in the duty of care protocol was reached.

**Table.** Study Characteristics

Source	Country	Population	Age at baseline, mean (SD), y	Total No. at baseline (female, %)	Intervention type (No. of modules) [duration]	Control condition	Measure of suicidal ideation	Dropout rate, %^a^	
								Intervention group	Control group
van Spijker et at,^33^ 2014	The Netherlands	Adults with suicidal thoughts	40.9 (13.7)	236 (66.1)	CBT, DBT problem-solving therapy, mindfulness-based therapy; unguided (6) [6 wk]	Wait-list	Beck Scale for Suicidal Ideation	9.5	8.3
Hill and Pettit,^34^ 2016	United States	School students with perceived burdensomeness	16.9 (1.7)	80 (68.8)	CBT; unguided (2) [2 wk] plus psychoeducational emails	Attention-control (emails with psychoeducational information)	Beck Scale for Suicidal Ideation	12.2	10.03
Hetrick et at,^35^ 2017	Australia	School students with suicidal ideation	14.7 (1.4)	50 (82.0)	CBT; guided (8) [10 wk] plus TAU	TAU (contact with the school well-being staff)	Suicidal Ideation Questionnaire	30.8	12.5
van Spijker et al,^36^ 2018	Australia	Adults with suicidal thoughts	40.6 (11.9)	418 (77.3)	CBT, DBT, problem-solving therapy, mindfulness-based therapy; unguided (6) [6 wk]	Attention-control (6-wk online modular lifestyle information program)	Intensity of Suicidal Ideation subscale of the Columbia Suicide Severity Rating Scale	43.5	48.3
Wilks et al,^37^ 2018	United States	Suicidal individuals with heavy episodic drinking and emotion dysregulation	38.0 (10.4)	59 (69.5)	DBT; guided (8) [8 wk]	Wait-list	Beck Scale for Suicidal Ideation	26.7	3.4
de Jaegere et al,^38^ 2019	Belgium	Adults with suicidal thoughts	35.7 (13.6)	724 (59.4)	CBT, DBT, problem-solving therapy, mindfulness-based therapy; unguided (6) [6 wk]	Wait-list	Beck Scale for Suicidal Ideation	74.0	52.1

Abbreviations: CBT, cognitive behavioral therapy; DBT, dialectical behavioral therapy; TAU, treatment as usual.

^a^ Rates shown are for participants who dropped out after the intervention was completed.

Three studies^33,36,38^ used the same intervention (“Living with deadly thoughts”) developed by van Spijker et al^39^ or an adapted version of it. This unguided intervention consisted of 6 modules, and participants were instructed to work for 30 minutes a day on each module during a 6-week period. The intervention had a strong focus on worry scheduling, aiming to help suicidal individuals to gain more control over their suicidal ideation. In addition, the intervention consisted of learning exercises and psychoeducation on emotion regulation, the identification and modification of automatic thoughts, and relapse prevention.^39^ Two interventions^34,35^ were designed for secondary school students. The 8 modules in the intervention by Hetrick et al^35^ included CBT elements such as behavioral activation, identification of automatic thoughts, problem solving, and cognitive restructuring. The intervention by Hill and Pettit^34^ consisted of 2 modules and included psychoeducation, exploration of situations in which adolescents perceive themselves as a burden, reality checking, and activity scheduling. One intervention^37^ was a dialectical behavioral therapy skills training consisting of 8 modules designed for heavy episodic drinkers experiencing suicidal ideation. The intervention focused especially on emotion dysregulation, which is associated with both suicide risk and binge drinking.^40^

### Risk of Bias

**Figure 2.**
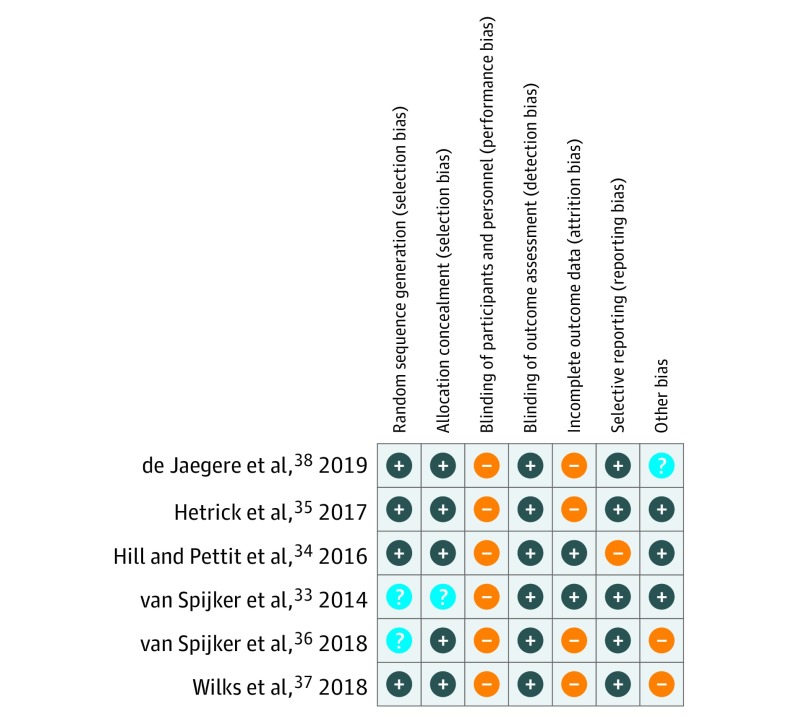
Risk of Bias Summary Ratings were performed using the Cochrane Risk of Bias Tool. Plus sign indicates low risk of bias; minus sign, high risk; and question mark, unclear.

An overview of the risk of bias assessment is presented in Figure 2. The largest source of potential bias resulted from the failure to blind participants. As is the case for most psychological intervention trials, blinding of participants was not possible. This is a potential risk of bias, in particular because all outcomes were self-reported. Substantial risk of bias was introduced by incomplete outcome data. Risk of attrition bias was high in 4 studies.^35,36,37,38^ One study did not provide robust estimates based on an imputation model or multilevel analysis for the meta-analysis^37^; 3 studies^35,36,38^ reported more than 20% dropout (up to 63.1%^38^) following the intervention, and in 2 of these,^35,38^ dropout was higher in the intervention group than in the control condition. Risk of bias was mostly low in the other evaluated categories.

### Meta-analysis

**Figure 3.**
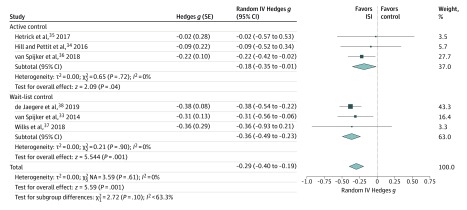
Between-Group Effects of Internet-Based Self-help Interventions (ISIs) on Suicidal Ideation Post Intervention Negative values indicate lower suicidal ideation in the intervention group compared with controls. Size of markers indicates weight. IV indicates inverse variance; and NA, not applicable.

All 6 studies reported effects on suicidal ideation and were included in the meta-analysis. The meta-analysis revealed a statistically significant effect following the intervention favoring ISI over controls (SMD, −0.29; 95% CI, −0.40 to −0.19; *P* < .001). Postintervention times ranged from 2 to 10 weeks after baseline. The forest plot is displayed in Figure 3. The overall level of heterogeneity was low (*I*^2^ = 0%).^32^

**Figure 4.**
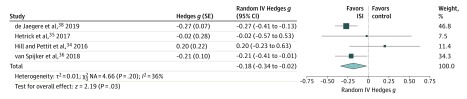
Between-Group Effects of Internet-Based Self-help Interventions (ISIs) on Suicidal Ideation at Follow-up Follow-up occurred 6 to 26 weeks after the postintervention period. Negative values indicate lower suicidal ideation in the intervention group compared with controls. Size of markers indicates weight. IV indicates inverse variance; NA, not applicable; and SE, standard error.

A follow-up comparison of suicidal ideation to 6 months post intervention was available from 4 studies,^33,34,35,36,38^ with assessment times ranging from 6 to 26 weeks after the postintervention period. At follow-up, the reduction of suicidal ideation was significant (SMD, −0.18; 95% CI, −0.34 to −0.02; *P* = .03) (Figure 4), and statistical heterogeneity was moderate (*I*^2^ = 36%).^32^

### Subgroup and Sensitivity Analyses

Subgroup and sensitivity analyses were performed for the effects post intervention. The subgroup analysis (Figure 3) for different types of control groups revealed an SMD of ISI compared with active controls of −0.18 (95% CI, −0.35 to −0.01; *P* = .04), whereas the SMD of ISI compared with wait-list controls was −0.36 (95% CI, −0.49 to −0.23; *P* < .001). Active controls received emails with psychoeducational content, were in contact with the school well-being staff, or received a modular lifestyle program that was matched to the ISI for suicide prevention. The variability between subgroups was *I*^2^ = 63.3. Sensitivity analyses showed a significant effect for ISI for adults in 4 studies^33,36,37,38^ (SMD, −0.32; 95% CI, −0.43 to −0.21; *P* < .001) and a significant effect for unguided ISI in 4 studies^33,34,36,38^ (SMD, −0.30; 95% CI, −0.41 to −0.20; *P* < .001).

An additional sensitivity analysis revealed that when the study by de Jaegere et al^38^ that had a weight of 43.3% in the meta-analysis post intervention was excluded, there was still a significant effect on suicidal ideation^33,34,35,36,37^ (SMD, −0.23; 95% CI, −0.37 to −0.09; *P* = .001). Furthermore, when trials at high risk of bias (ie, >2 items of the Cochrane Risk of Bias Tool rated at high risk of bias) were excluded,^36,37^ a comparable effect size to the overall effect post intervention was found^33,34,35,38^ (SMD, −0.32; 95% CI, −0.45 to −0.20; *P* < .001).

### Secondary Outcomes

Regarding completed suicides, 2 studies^33,38^ included anecdotal reports that no suicide death occurred during the study. However, both studies did not include assessment of suicide deaths in the methods section and did not specifically declared it as an outcome, so how they ensured that suicide deaths could be detected remains unclear. Two studies^35,36^ reported on suicide attempts, and both trials did not find an effect. A total of 5 studies^33,34,35,36,38^ reported data on depressive symptoms, whereas 4 studies^33,35,36,38^ reported data on anxiety and hopelessness symptoms. Effect sizes and 95% CIs for depressiveness, anxiety, and hopelessness are displayed in eTable 1 in the Supplement. Most of the trials found almost no significant effects, with the exception of 1 study^38^ that found significant reductions of depressive symptoms, anxiety, and hopelessness across all time points with small to medium effect sizes.

Adherence to the intervention was reported in 5 studies,^33,34,35,36,37^ using a variety of definitions, so that we could only summarize data from 3 trials.^33,34,37^ A total of 45.2% to 92.7% of participants completed at least half of the modules, with a mean of 64.6%. The percentage of participants completing no modules ranged from 6.5% to 22.4%, with a mean of 12.1%.

### Unpublished Studies

We searched for registered trials and study protocols to check for potential publication bias, because unpublished trials might be different from published trials, and to give an overview of ongoing trials. The search of ongoing trials in ClinicalTrials.gov yielded 313 records. No completed but unpublished trials were identified. In addition, we identified 9 relevant study protocols in the database searches. For 4 protocols, subsequent publications were identified and included in this review.^33,35,36,37^ For 5 protocols, we could not identify any publication of results; however, it could be presumed that trials or publication processes were still ongoing, because the identified protocols were published from 2016 to 2018.^41,42,43,44,45^ Thus, we did not find any evidence of publication bias. The database and clinical trial register searches revealed a total of 7 potentially relevant ongoing trials that are reviewed in eResults in the Supplement.

### Quality of Evidence

The quality of evidence was rated for the effect on suicidal ideation post intervention and at follow-up by using the GRADE procedure^32^ (eTable 2 in the Supplement). Ratings indicated a moderate quality of evidence for the reduction in suicidal ideation scores post intervention. The main reason for downgrading was risk of bias resulting from high attrition rates, the failure to blind participants, and the use of wait-list controls. Quality of evidence was rated very low at follow-up because only 4 trials^34,35,36,38^ provided follow-up data. The confidence in the effect estimate was additionally limited by exceedingly high dropout rates (>50% overall), by 1 trial finding an effect in favor of the control condition,^34^ because of variance in assessment timing, and because of wide 95% CIs (−0.34 to −0.02) indicating a high degree of uncertainty.

## Discussion

This report is, to our knowledge, the first systematic review and meta-analysis to evaluate whether iCBT interventions that directly target suicidality are associated with reductions in suicidal ideation. Six RCTs were identified and included in this meta-analysis, of which 1 was a pilot trial.^34^ Available data suggest that ISIs significantly reduced suicidal ideation post intervention and provide preliminary evidence that this effect can be maintained at follow-up. Statistical and clinical heterogeneity was low. The identified effect size post intervention is similar to those found in meta-analyses for face-to-face CBT for suicidal ideation^24^ and for interventions for youth that directly target suicidality,^46^ suggesting that iCBT may be a useful alternative to traditional therapies.

The GRADE rating indicated a moderate quality of evidence for the effect post intervention. Subgroup analysis revealed higher effect sizes in wait-list–controlled compared with actively controlled study designs, which is a common finding in meta-analyses.^47,48^ Wait-list controls might introduce a nocebo effect, leading to a possible overestimation of effects due to a worsening of symptoms in the control condition.^49^

Taking into account that ISIs are highly scalable^15,50^ and that suicidal ideation is a major risk factor for suicide,^1^ even small effects on suicidal ideation might have substantial effects when implemented at scale. Ideally, in addition to ISIs being made available online, they could be integrated into health services, such as through prescription by clinicians as standalone or adjunctive therapies.^51^ Moreover, crisis services, such as the National Suicide Prevention Lifeline, could be complemented by iCBT. Above that, multiple implementation pathways, such as search engine–based approaches, should be explored to identify optimal distribution channels to reach individuals at risk of suicide.^52^

The participants of the studies in this meta-analysis were predominantly female, which is in line with the higher prevalence of suicidal ideation^1,4^ and help-seeking behavior in women.^53^ Rates of suicide have been higher in men than in women; however, this gender gap is narrowing.^54^ The field should move forward by developing tailored interventions to address the specific needs of individual users, instead of distributing one-size-fits-all interventions. Using machine-learning algorithms might be a viable way to reach this goal.^55^ New technological opportunities, such as digital phenotyping, could be used to perform risk assessments and to personalize interventions to the current needs of individuals at risk of suicide.^55,56,57,58^

Furthermore, future research could strengthen the evidence base of ISIs for suicide prevention by conducting larger-scale effectiveness trials with active controls or noninferiority trials and by including longer follow-up measurement occasions. These approaches are not only needed to increase confidence in the effectiveness of ISIs but also to examine the potential effect of these interventions on suicide behavioral outcomes.^59,60^ Fortunately, the field appears to be moving in this direction, with 2 large-scale, ongoing trials planned to evaluate the effectiveness of internet-based interventions on suicidal behavior.^41,45^ A possible way to strengthen the effects of ISI could be to undertake implementation trials of strategies to improve uptake and adherence, which has been suboptimal in previous studies.^38^ Adding human support could contribute to achieve this goal, because guided interventions typically demonstrate larger effects than unguided interventions and might increase adherence.^61,62^ However, adding guidance limits the scalability and anonymity to some extent. To make full use of the existing date and to identify moderators and mediators of effects, an individual patient data meta-analysis could also be a fruitful next step.

### Limitations

This systematic review and meta-analysis has some limitations. First, subgroup and sensitivity analyses need to be interpreted with caution, because they rely on observational data. Owing to the low number of included studies (n = 6), results of subgroup and sensitivity analyses are likely to be confounded with other variables.^28^ Second, although statistical heterogeneity was low (*I*^2^ = 0%), there are most likely differential effects for different interventions, populations, or designs that could not be detected owing to the imprecision of effect estimates. Third, it remains largely unclear whether reductions in suicidal ideation can be maintained. The GRADE rating indicated a very low quality of evidence at follow-up, because the estimate is based on data from only 4 studies with high dropout rates, and follow-up assessment times ranged from 6 to 26 weeks. Finally, publication bias could not be ruled out.^28^ However, we did not find any evidence for the presence of publication bias by screening the trial register and study protocols. Furthermore, included studies consist not only of studies with significant effects but also small trials with nonsignificant results, which might be less likely to be published.^63^

## Conclusions

The current body of research suggests that iCBT may be an important part of future suicide prevention efforts. Owing to their high scalability, the implementation of iCBT interventions into health systems and communities might translate to a substantial effect on reducing the disease burden of suicidal ideation. However, the field is still in its infancy, and continued robust research efforts are needed to confidently establish the effectiveness of interventions, identify moderators and mediators, and explore pathways to deliver tailored interventions for individuals at risk of suicide.

## Notes

Supplement. **eMethods.** Amendments to the Study Protocol **eTable 1.** Between-Groups Effect Sizes of Secondary Outcomes **eTable 2.** GRADE Ratings for Suicidal Ideation **eResults.** Results of Ongoing Trials **eReferences.**
