# Supplementary material for: Internet-Based Cognitive Behavioral Therapy to Reduce Suicidal Ideation: A Systematic Review and Meta-analysis
Source: JAMA Netw Open. 2020 Apr 28;3(4):e203933. doi: 10.1001/jamanetworkopen.2020.3933 (PMC7189224; doi:10.1001/jamanetworkopen.2020.3933)
Supplement: Supplement. — eMethods. Amendments to the Study Protocol eTable 1. Between-Groups Effect Sizes of Secondary Outcomes eTable 2. GRADE Ratings for Suicidal Ideation eResults. Results of Ongoing Trials eReferences. [file jamanetwopen-3-e203933-s001.pdf]

Supplementary Online Content

Büscher R, Torok M, Terhorst Y, Sander L. Internet-based cognitive behavioral therapy to reduce suicidal ideation: a systematic review and meta-analysis. *JAMA Netw Open*. 2020;3(4):e203933. doi:10.1001/jamanetworkopen.2020.3933

eMethods. Amendments to the Study Protocol
eTable 1. Between-Groups Effect Sizes of Secondary Outcomes
eTable 2. GRADE Ratings for Suicidal Ideation
eResults. Results of Ongoing Trials
eReferences.

This supplementary material has been provided by the authors to give readers additional information about their work.

©2020 Büscher R et al. *JAMA Network Open*.

eMethods. Amendments to the Study Protocol

Amendment 1:

We did not assess publication bias by using a funnel plot.

Reason: The number of studies was considered to be too low.

Amendment 2:

We did not conduct the pre-planned subgroup analyses for guidance and age of target group. Instead, we conducted two sensitivity analyses by excluding guided interventions and interventions for youth.

Reason: The total number of participants included in the subgroups of guided and youth interventions was considered to be too low (n=109 and n=130, respectively).

©2020 Büscher R et al. *JAMA Network Open*.

eTable 1. Between-Groups Effect Sizes of Secondary Outcomes

		Hopelessness		Depressiveness		Anxiety	
**First author (year)**	***N***	**Measure**	**d [95% CI]**	**Measure**	**d [95% CI]**	**Measure**	**d [95% CI]**
van Spijker (2014)							
Post (6 weeks)	236	BHS	-0·28 [-0·54; -0·03]	BDI-II	-0·22 [-0·48; 0·03]	HADS-A	-0·14 [-0·40; 0·11]
Hill (2016) [a]							
Post (2 weeks)	80	n.a.	n.a.	RADS-2 (short form)	-0·29 [-0·73; 0·15]	n.a.	n.a.
Follow-up (8 weeks)	80		n.a.		-0·42 [-0·86; 0·03]		n.a.
Hetrick (2017) [a, b]							
Post (2 weeks)	39	BHS	-0·02 [-0·65; 0·61]	RADS-2	-0·20 [-0·84; 0·43]	MASC	-0·35 [-0·98; 0·29]
Follow-up (8 weeks)	30		-0·11 [-0·83; 0·61]		-0·45 [-1·18; 0·28]		-0·31 [-1·03; 0·42]
van Spijker (2018) [a,c]							
Post (6 weeks)	418	Burns Hope-lessness Scale	0·17 [-0·02; 0·37]	CES-D	-0·07 [-0·26; 0·13]	GAD-7	0·06 [-0·13; 0·25]
Follow-up (7,5 months)	418		0·03 [-0·16; 0·22]		0·08[-0·11;0·27]		0·23 [0·04; 0·42]
Follow-up (13,5 months)	418		-0·08 [-0·28; 0·11]		-0·20 [-0·39; 0·00]		-0·33 [-0·52; -0·14]
de Jaeagere (2019)							
Post (6 weeks)	724	BHS	-0·46 [-0·61; -0·31]	BDI-II	-0·38 [-0·52; -0·23]	HADS-A	-0·37 [-0·52; -0·22]
Follow-up (12 weeks)	724		-0·53 [-0·68; -0·39]		-0·44 [-0·59; -0·29]		-0·38 [-0·52; -0·23]

Note: Negative effect sizes indicate reductions in symptom severity
[a] Effects calculated based on reported means, standard deviations and number of participants
[b] No ITT analysis
[c] Effect sizes of mean changes from baseline were calculated due to baseline differences in suicidal ideation
n.a.: Outcome was not assessed
BHS: Beck Hopelessness Scale
BDI-II: Beck Depression Inventory
CES-D: Centre for Epidemiological Studies Depression Scale
RADS-2: Reynolds Adolescent Depression Scale-2
HADS-A: Hospital Anxiety and Depression Scale

©2020 Büscher R et al. *JAMA Network Open*.

eTable 2. GRADE Ratings for Suicidal Ideation

GRADE rating for n = 6 studies reporting self-rated severity of suicidal ideation at post-intervention

GRADE criteria	Rating	Reasons for down- or upgrading	Quality of the evidence
Study design	RCT: high quality		
Risk of Bias	Serious limitations (-1)	Participants not blinded. 3 out of 6 studies with critical dropout-rates (>20%; 2 studies > 40%). Waitlist controls might have overestimated effect size	
Inconsistency	No serious limitations	Small effect size (SMD = -0·29), I^2 = 0%. Results in the same direction	
Indirectness	No serious limitations	Population limited to individuals with suicidal ideation.	⊕⊕⊕⊖
Imprecision	No serious limitations	More than 1500 participants in total; pooled effect size and CIs all consistent with small effects; absence of any outlier suggests low imprecision, despite small sample sizes and the fact that studies generally were underpowered	
Publication Bias	Undetected (insufficient number of studies)	No evidence in trial register search. Significant, non-significant and large trials reported.	

Note. ⊕⊕⊕⊕ = high, ⊕⊕⊕⊖ = moderate, ⊕⊕⊖⊖ = low, ⊕⊖⊖⊖ = very low. Included studies: Hetrick et al., 2017; Hill & Pettit, 2016; Jaegere et al., 2019; van Spijker et al., 2018; van Spijker et al., 2014; Wilks et al., 2018.

GRADE rating for n = 4 studies reporting suicidal ideation at follow-up (up to 6 weeks after post)

GRADE criteria	Rating	Reasons for down- or upgrading	Quality of the evidence
Study design	RCT: high quality		
Risk of Bias	Serious limitations (-1)	Participants not blinded. 3 out of 4 studies with critical dropout-rates (>20%; 2 studies >60%). Waitlist controls might have overestimated effect size	
Inconsistency	Serious limitations (-1)	Moderate statistical heterogeneity (I^2 = 36%); results are not all in the same direction (Hill favors control); varying assessment times (6 – 26 weeks after post)	
Indirectness	No serious limitations	Population limited to individuals with suicidal ideation	⊕⊖⊖⊖
Imprecision	Serious limitations (-1)	More than 1200 participants in total (ITT), but only n=4 studies provided follow-up data; very high dropout (<500 participants raw); 95% CI (-0.34, -0.02) indicates a high degree of uncertainty	
Publication Bias	Undetected (insufficient number of studies)	No evidence in trial register search. Significant, non-significant and large trials reported.	

Note. ⊕⊕⊕⊕ = high, ⊕⊕⊕⊖ = moderate, ⊕⊕⊖⊖ = low, ⊕⊖⊖⊖ = very low. Included studies: de Jaegere et al., 2019; van Spijker et al., 2018; Hill & Pettit, 2016; Hetrick et al., 2017.

eResults. Review of Ongoing Trials

We identified two large population-based trials that focus on the effectiveness of internet-based interventions on suicide attempt and suicide. This included a web- and mobile-based intervention (NCT03565562[1]) and an internet-based skills training based on dialectical behavioral therapy (NCT02326883[2]), with 100,000 and 19,500 expected participants, respectively. The remaining five studies were planned on a smaller scale and mainly focused on the effectiveness of digital interventions on suicidal ideation. Two ongoing trials planned to examine the effectiveness of the intervention by van Spijker et al.,[3] adapted for Turkish migrants,[4] and translated to Danish (NCT02872610[5]). One trial planned to use a web- and mobile-based platform providing information, self-monitoring, counselling and a virtual community for school students in Chile (NCT03514004[6]). Finally, we identified one trial examining a computerized intervention for active military personnel (NCT03571750), and one trial with two internet-based programs for the prevention of suicidal ideation in medical students (NCT03949556).

eReferences.

1. Coralie G, Kathleen T, Marie-Amelie V, Anais LJ, Hazo JB, Karine C. Printemps project: e-health intervention for suicide prevention and mental health promotion in the French general population. *Eur psychiatry*. 2018;48:S222-S223. doi:10.1016/j.eurpsy.2017.12.016

2. Simon GE, Beck A, Rossom R, et al. Population-based outreach versus care as usual to prevent suicide attempt: study protocol for a randomized controlled trial. *Trials*. 2016;17(1):452. doi:10.1186/s13063-016-1566-z

3. van Spijker BAJ, van Straten A, Kerkhof AJFM. The effectiveness of a web-based self-help intervention to reduce suicidal thoughts: a randomized controlled trial. *Trials*. 2010;11:25. doi:10.1186/1745-6215-11-25

4. Eylem O, van Straten A, Bhui K, Kerkhof AJFM. Protocol: Reducing suicidal ideation among Turkish migrants in the Netherlands and in the UK: effectiveness of an online intervention. *Int Rev Psychiatry*. 2015;27(1):72-81. doi:10.3109/09540261.2014.996121

5. Mühlmann C, Madsen T, Hjorthøj C, Kerkhof A, Nordentoft M, Erlangsen A. The Self-help Online against Suicidal thoughts (SOS) trial: study protocol for a randomized controlled trial. *Trials*. 2017;18(1):45. doi:10.1186/s13063-017-1794-x

6. Mascayano F, Schilling S, Tapia E, et al. Using information and communication technologies to prevent suicide among secondary school students in two regions of Chile: a randomized controlled trial. *Front psychiatry*. 2018;9(236):1-9. doi:10.3389/fpsyt.2018.00236
